# Are hematopoietic cell transplant recipients with Gram‐negative bacteremia spending more time outpatient while on intravenous antibiotics? Addressing trends over 10 years at a single center

**DOI:** 10.1002/iid3.486

**Published:** 2021-07-21

**Authors:** Margaret L. Lind, Steven Roncaioli, Catherine Liu, Andrew Bryan, Ania Sweet, Frank Tverdek, Mohamed Sorror, Amanda I. Phipps, Steven A. Pergam

**Affiliations:** ^1^ Vaccine and Infectious Disease Division Fred Hutchinson Cancer Research Center Seattle Washington USA; ^2^ Department of Epidemiology University of Washington School of Public Health Seattle Washington USA; ^3^ Infection Control, Department of Medicine BronxCare Health System New York New York USA; ^4^ Department of Medicine University of Washington School of Medicine Seattle Washington USA; ^5^ Clinical Research Division Fred Hutchinson Cancer Research Center Seattle Washington USA; ^6^ Antimicrobial Stewardship and Outpatient Parenteral Antimicrobial Therapy Programs Seattle Cancer Care Alliance Seattle Washington USA; ^7^ Department of Laboratory Medicine University of Washington Seattle Washington USA; ^8^ Department of Pharmacy University of Washington Seattle Washington; ^9^ Public Health Sciences Division Fred Hutchinson Cancer Research Center Seattle Washington USA

**Keywords:** ambulatory care, antibiotic stewardship, Gram‐negative rod bacteremia, hematopoietic cell transplant, outpatient care

## Abstract

**Introduction:**

The increasing proportion of outpatient allogeneic hematopoietic cell transplants (HCTs) coupled with increased access of once‐daily broad‐spectrum antibiotics and evidence that outpatient antibiotic treatment may be safer and less costly than inpatient treatment, suggest that allogeneic HCT recipients with Gram‐negative rod bacteremia (GNRBs) are increasingly being treated in ambulatory care settings.

**Methods:**

Using data from the first GNRB event that occurred within the first 100 days posttransplantation among allogeneic HCT recipients transplanted at a single center between 2007 and 2016, we estimated the temporal trends in GNRB incidence and treatment management of GNRBs and identified if patient or infection characteristics impacted observed trends.

**Results:**

A total of 11% (238/2165) of the observed allogeneic HCT recipients experienced ≥1 GNRB with available resistance data and contributed antibiotic treatment time. Patients, on average, received 55.1% of their antibiotic treatment in an outpatient setting and we observed a significant decline in the proportion of treatment time spent outpatient (crude: −3.3% [95% confidence interval: −5.0, −1.6%]). We observed similar declines in the proportion of treatment time spent outpatient among patients with similar GNRB and pretransplant complexity factors but not among patients with similar posttransplant complications (*p* value: .165).

**Conclusion:**

These results suggest that, despite increased availability of outpatient suitable treatment options, allogeneic HCT recipients with GNRBs received less treatment in outpatient settings. However, among patients with similar posttransplant complications, the lack of significant decline suggests that treatment location decisions remained consistent for patients with similar posttransplant complications. These findings suggest the need for additional interventions targeting outpatient antibiotic treatment among allogeneic HCT recipients with GNRBs.

## Graphical abstract

Over a 10‐year period, Gram‐negative rod bacteremia (GNRB) incidence and the proportion of GNRBs treatment time spent outpatient declined significantly. However, among patients with similar posttransplant complications, no decline in outpatient treatment was observed. This suggests that the decline in outpatient antibiotic days may be linked to increased frequency of posttransplant complications.

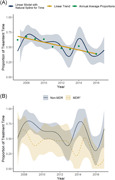

## 1 INTRODUCTION

Gram‐negative rod bacteremia (GNRB), a leading cause of mortality among allogeneic hematopoietic cell transplant (HCT) recipients, has, historically, been treated with inpatient intravenous (IV) antibiotics.^1^ However, evidence shows that inpatient antibiotic administration may be associated with higher healthcare costs and risk of adverse outcomes when compared to outpatient delivery.^2^, ^3^, ^4^ The evidence of such associations, coupled with the availability of once‐daily broad‐spectrum antibiotics, such as ertapenem, and an overall trend toward outpatient HCT care supports the idea that HCT recipients are increasingly being treated for GNRBs in ambulatory care settings.^2^, ^3^, ^4^ To examine if HCT recipients with GNRBs are, in fact, receiving more care in outpatient settings, we evaluated if the proportion of targeted antibiotic treatment time allogeneic HCT recipients with GNRBs in ambulatory settings changed over a 10‐year period among all patients and among patients with similar pretransplant complexities, GNRB complexity, and posttransplant complications. This analysis was conducted on a large sample of adult allogeneic HCT recipients receiving care over a 10‐year period from a single center.

## 2 METHODS

### 2.1 Data collection and cohort development

We performed a retrospective review of adult (≥18 years) allogeneic HCT recipients with GNRB from Seattle Cancer Care Alliance/Fred Hutchinson Cancer Research Center (SCCA/FHCRC) who were transplanted between January 1st, 2007 and December 31st, 2016. We restricted our analysis to first culture confirmed GNRB within the first 100 days posttransplantation and, in alignment with center standards, assumed patients received 14 days of antibiotics; at death, the number of days was equal to the number they survived on antibiotic therapy. To be conservative, given the time between blood culture collection and organism identification, we defined targeted antibiotic administration start date as 2 days after collection. All data were extracted from a center‐maintained, prospectively‐collected database. The FHCRC institutional review board approved this study.

### 2.2 Transplant procedures

Conditioning regimens of myeloablative intensity included mostly cyclophosphamide combined with either at least 12 Gy TBI or busulfan (levels targeted to plasma mean steady‐state concentrations of 800–900 ng/ml)^5^; most were given methotrexate/CSP for graft‐versus‐host disease (GVHD) prophylaxis.^6^ All recipients of myeloablative regimens were hospitalized for 3–4 weeks before being discharged to the outpatient clinic.

Regimens of reduced‐intensity or nonmyeloablative conditioning intensity were generally offered to patients who were either 50 or older or, if younger than 50, had significant preexisting medical problems or had failed high‐dose autologous HCT. Recipients of these regimens were generally treated in the outpatient clinic during the first 100 days before returning to their referring physicians and were admitted to the hospital only as required for treatment of complications.

Diagnoses and clinical grading of acute and chronic GVHD were performed using standard criteria.^7^, ^8^ Primary treatment of GVHD consisted of systemic corticosteroids, oral beclomethasone with or without systemic corticosteroids, or reinstitution of CSP.

Patients and donors were matched for HLA‐A, ‐B, and ‐C antigens by either intermediate resolution DNA typing (to a level at least as sensitive as serology) or by high‐resolution techniques. Patients and donors were matched for HLA‐DRB1 and DQB1 alleles.^9^ All patients received infection prophylaxis according to standard institutional guidelines.^10^, ^11^, ^12^, ^13^

### 2.3 Antibiotic and culture practices

Cultures were collected at the discretion of the treating team; however, center‐specific guidelines recommended that two sets of blood cultures be drawn for patients with fever or other signs of infection. Additionally, single‐set, routine surveillance blood cultures were collected bi‐weekly while inpatient or weekly while outpatient among patients treated for GVHD.^14^ Levofloxacin was used as first‐line neutropenic prophylaxis. “Although regimens varied over time, piperacillin/tazobactam or a carbapenem were most frequently the initial antibiotic therapy of choice for enteric GNRBs, and ceftazidime/cefepime for non‐enteric GNRBs.”

### 2.4 Factor definitions

Our primary outcome of interest was the proportion of treatment days spent in the outpatient setting ([outpatient treatment days]/14) and we defined outpatient days as any day with partial outpatient care delivery. We identified the following a priori posttransplant complications: severe acute GVHD (overall Grade >3),^15^ neutropenia and location (inpatient vs. outpatient) at time of GNRB diagnosis; inpatient location was included as a proxy for additional posttransplant complications. Patients were considered neutropenic until they had a neutrophil count of more than 500 cells/μl for 3 consecutive days; both neutropenia and GVHD were considered time varying covariates. Additionally, we selected the following GNRB complexity factors (multidrug resistance [MDR], fluoroquinolone resistance [FR], polymicrobial culture, presence of hard‐to‐treat organism [those requiring multiple IV doses or drugs daily], and days to infection onset) and pretransplant complexity factors (age at transplant, donor relationship [unrelated vs. related], HCT‐specific Comorbidity Index [HCT‐CI],^16^ conditioning regimen [myeloablative vs. nonmyeloablative], and underlying disease) a priori.^17^, ^18^

We defined MDR as organisms with intermediate or full resistance to ≥1 agent in ≥3 antibiotic classes: cephalosporins, penicillin/antipseudomonal penicillin + beta‐lactamase inhibitors, carbapenems, aminoglycosides, and fluoroquinolones; all *Stenotrophomonas maltophilia* isolates were considered MDRO.^19^, ^20^ Polymicrobial GNRBs were considered MDR if any organism met criteria. Antibiotic resistance was determined using the clinically reported susceptibility interpretations at the time of result reporting. We defined days to infection as the number of days between transplantation and culture collection. Hard‐to‐treat infections, for which there is an absence of once daily treatment options, were defined as the presence of a carbapenem resistant Enterobacteriaceae species or fluoroquinolone resistant *Pseudomonas aeruginosa, Acinetobacter* species, or *Stenotrophomonas maltophilia*.

### 2.5 Statistical analysis

We estimated the average yearly change in GNRB, FR‐GNRB, and MDR‐GNRB incidence using Poisson regressions with continuous year variables. We defined patient days at risk as days between transplant and death, first GNRB or 100‐day posttransplant follow‐up, whichever came first. We estimated the magnitude and direction of the association between calendar year and proportion of time spent outpatient using linear regression with a continuous year variable. With a natural spline for time with knots at each year between 2008 and 2016, we estimated the shape of the outpatient treatment time trend.

In an exploratory analysis, we examined if the portion of time spent outpatient among patients with similar pretransplant and GNRB complexities and posttransplant complications changed over time using complete case, multivariable linear regressions. To ensure the stability of our confidence intervals (CI) in the presence of possible collinearity, we calculated variance inflation factors (VIFs) and found that no factors exceeded a VIF of 2.5.^21^ Additionally, we evaluated if MDR modified the temporal trend by comparing a generalized additive model with an interaction between the natural spline for date of infection and MDR status to the model minus the interaction using a likelihood ratio test. Additionally, we estimated the average time to infection onset by year. Finally, in a post hoc analysis, we examined the temporal trends for individual factor components of adjustment models with a differing result than the unadjusted model. The linear change in time was estimated using a Poisson Regression with a continuous year variable and, for visual purposes only, the shape of the time trend was estimated using a Poisson Regression with a natural spline with knots at each year of follow‐up. All analyses were performed using the R base and mgcv packages.^22^, ^23^

## 3 RESULTS

**Figure 1:**
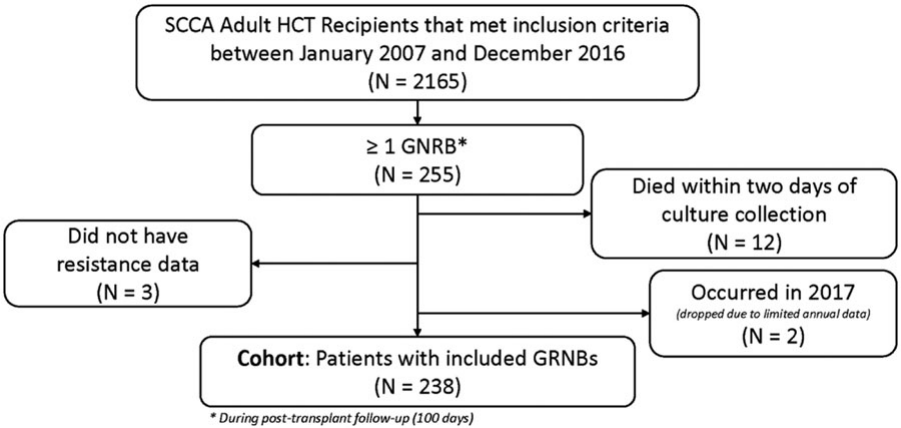
Flowchart of adult allogeneic hematopoietic cell transplant recipient inclusion Legend: Flowchart of hematopoietic cell transplant (HCT) recipient study population. Gram‐negative rod bacteremia (GNRB) was identified using blood culture confirmation and we limited the analysis to first GNRB during patient follow‐up (first 100 days posttransplant). A total of 17 individuals were dropped from the analysis, 12 died before our assumed targeted antibiotic start date, 2 had their first GNRB in 2017, and 3 did not have resistance data

Of the 2165 SCCA patients who received a transplant between January 1, 2007 and December 31st, 2016, 243 (11.2%) experienced ≥1 GNRB during follow‐up and contributed antibiotic treatment time (survived ≥2 days following culture collection). We dropped five individuals due to unavailable resistance data and late GNRBs (occurring in 2017) and our final cohort consisted of 238 individuals (Figure 1). Among these recipients, the median age at diagnosis was 53 years (interquartile range [IQR]: 43, 60) and the most prevalent underlying condition was acute myeloid leukemia. The median time in days between transplant and first GNRB event was 51.0 (IQR: 14.0, 75.8) days, 81 events (34.0%) occurred before engraftment, and the median HCT‐CI was 7 (IQR: 4, 8). Recipients who received more than 50% treatment inpatient were more likely to have neutropenia (43.1% vs. 11.8%) and severe acute GVHD (35.3% vs. 10.3%) at the time of GNRB and have a shorter median time till first GNRB (35 vs. 57 days) than patients who received more than 50% care outpatient (Table 1). Among patients with severe acute GVHD at time of first GNRB event, 37 (74.0%) had a gut GVHD score of 3 or more and 25 (50.0%) had a skin GVHD score of 3 or more. Of the 50 GNRB events identified in patients with severe acute GVHD, 22 (44.0%) were identified through surveillance cultures.

**Table 1:** Characteristics of adult allogeneic HCT recipients transplanted between January 2007 and December 2016 with lab‐confirmed Gram‐negative rod bacteremia by majority antibiotic treatment setting

	Total (*n* = 238)	Majority outpatient treatment (*n* = 136)^a^	Majority inpatient treatment (*n* = 102)^b^
Age (years)—median, 1st–3rd Quartile	53 (43–60)	51 (42–59)	54 (45–61)
Male—*N*, %	117 (49.2)	64 (47.1)	53 (52.0)
Race/ethnicity—*N*, %			
Asian/Pacific Islander	16 (6.7)	8 (5.9)	8 (7.8)
Black	8 (3.4)	4 (2.9)	4 (3.9)
Caucasian	169 (71.0)	97 (71.3)	72 (70.6)
Hispanic	20 (8.4)	12 (8.8)	8 (7.8)
Other	25 (10.5)	15 (11.0)	10 (9.8)
Underlying disease—*N*, %			
Acute lymphoid leukemia	37 (15.5)	21 (15.4)	16 (15.7)
Acute myeloid leukemia	84 (35.3)	45 (33.1)	39 (38.2)
Myelodysplastic syndromes	41 (17.2)	15 (11.0)	26 (25.5)
Multiple myeloma	12 (5.0)	10 (7.4)	2 (2.0)
Non‐Hodgkin lympoma	27 (11.3)	18 (13.2)	9 (8.8)
Other	37 (15.5)	27 (19.9)	10 (9.8)
Unrelated donor—*N*, %	159 (66.8)	83 (61.0)	76 (74.5)
Comorbidity Index (HCT‐CI)—median, IQR*	7 (4–8)	7 (6–8)	7 (4–8)
Severe Acute GVHD (≥Grade 3)—*N*, %	50 (21.0)	14 (10.3)	36 (35.3)
Polymicrobial bacteremia—*N*, %	23 (9.7)	17 (12.5)	6 (5.9)
Hard‐to‐treat organism—*N*, %	9 (3.8)	2 (1.5)	7 (6.9)
Myeloablative conditioning regiment—*N*, %	90 (37.8)	48 (35.3)	42 (41.2)
Neutropenia (<500 cells/ml)—*N*, %	60 (25.2)	16 (11.8)	44 (43.1)
Days till first GNRB—median, IQR	51 (14–756)	57 (34–80)	35 (7–62)

*Note*: *Data limited to the 199 individuals with scores.

Abbreviations: GVHD, Graft‐versus‐host disease; HCT, hematopoietic cell transplant; IQR, interquartile range.

^a^ Defined people who spent more than 7 of their 14 days outpatient.

^b^ Defined people who spent more than 7 of their 14 days inpatient.

*Escherichia coli* was the most frequently observed GNRB species and made up 21.1% (50/238) of total events. After *E. coli, Pseudomonas* was the most frequently observed genus and made up 17.2% (41/238) of GNRBs. *Pseudomonas* was followed closely by *Klebsiella*, which accounted for 16.8% (40/238) of the total GNRB events. In total 23 (9.7%) of the 238 events were polymicrobial and 79 (33.2%) were classified as multidrug resistant.

**Figure 2:**
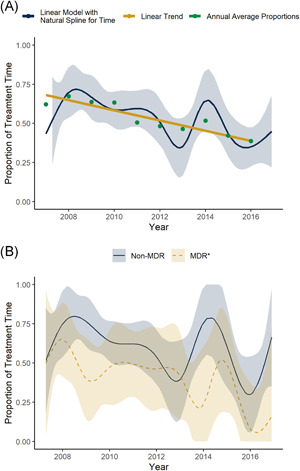
Proportion of antibiotic treatment time hematopoietic cell transplant (HCT) recipients with Gram‐negative rod bacteremia (GNRB) spent in ambulatory care settings over a 10‐year period. Legend: (A) Average proportion of time allogeneic HCT recipients with GNRB received targeted antibiotic treatment in ambulatory care settings. Annual average proportion of time spent receiving antibiotics in outpatient settings (turquoise points), the continuous time trend of the average proportion of time spent receiving antibiotics in outpatient settings with 95% confidence intervals (navy curve with ribbon), and the linear time trend of the annual average proportion of time spent receiving antibiotics in outpatient settings (gold line). (B) Average proportion of time allogeneic HCT recipients with multi‐drug resistant (MDR) and non‐MDR GNRB received targeted antibiotic treatment in ambulatory care settings. MDR defined as cephalosporins, penicillin/anti‐pseudomonal penicillin + beta‐lactamase inhibitors, carbapenems, aminoglycosides, and fluoroquinolones; or any *Stenotrophomonas maltophilia* isolate

The incidence of GNRB and MDR‐GNRB decreased by 7.0% (95% CI: −11.1, −2.7%) and 6.8% (CI: −13.9, 0.8%) on average per year, respectively. Conversely, the incidence of FR‐GNRB increased by 1.3% (CI: −5.9, 9.1%) on average. We did not observe any significant linear change in the average age of transplant or HCT‐CI by year of follow‐up (Table S1). We also found no change in the percentage of non‐Hodgkin's Lymphoma cases undergoing transplant. Over the study period, patients received an average of 55.1% (CI: 50.1, 60.1%) of their GNRB treatment outpatient. We observed a nonlinear decline in the proportion of time spent outpatient following GNRB diagnosis (Figure 2A). The unadjusted linear trend was found to be statistically significant (*p* value: <.001) and each year the average proportion of time spent in the outpatient setting dropped by 3.3% (CI: −5.0, −1.6%). The significance held following adjustment for pretransplant complexity (*p* = .015) and GNRB complexity (*p* = .003) but not following adjustment for posttransplant complications (*p* = .165) or when fully adjusted (*p* = .608) (Table 2); 39 recipients had no HCT‐CI and were dropped from the pretransplant and fully adjusted models. From our post‐hoc analysis of the individual factors included in the posttransplant complications adjusted model, we found that the proportion of GNRBs identified during inpatient stays and the proportion identified while patients were neutropenic increased annually on average (Table S2). However, the true increase was likely nonlinear (Figure S1).

**Table 2:** Difference in the average percent of time spent receiving antibiotics in the outpatient setting following positive GNRB culture result

	Difference in average percent spent outpatient	95% Confidence interval	*p* Value
Crude	−3.3%	(−5.0, −1.6%)	<.001
Factor adjusted models			
GNRB complexity^a^	−2.5%	(−4.1, −0.9%)	.003
Posttransplant complications^b^	−1.4%	(−3.4, 0.6%)	.165
Pretransplant complexity factors^c^	−2.7%	(−4.9, −0.5%)	.015
Fully adjusted model^d^	−0.7%	(−3.5, 2.1%)	.608

^a^ Adjusted for binary indicator of multidrug resistance, fluoroquinolone resistance, polymicobial culture, presence of hard to treat organism, and time to culture collection.

^b^ Adjusted for severe GVHD (Grade 3 or more), neutropenia (500 cells/µl or less) at time of GNRB diagnosis, and inpatient at time of GNRB diagnosis.

^c^ Adjusted for age at transplant, donor type, HCT specific comorbidity index (HCT‐CI), conditioning regimen (myeloablative or nonmyeloablative, and underlying disease).

^d^ Adjusted for age at transplant, donor type, HCT specific comorbidity index (HCT‐CI), underlying disease, severe GVHD (Grade 3 or more), neutropenia (500 cells/µL or less) at time of GNRB diagnosis, conditioning regimen (myeloablative or nonmyeloablative, inpatient at time of GNRB diagnosis, polymicrobial indicator, and binary indicators of fluoroquinolone, and multidrug resistance)

GNRBs were most frequently resistant to at least one penicillin/antipseudomonal penicillin + beta‐lactamase inhibitors (69.7% [166/238]; percent resistant to Piperacillin‐Tazobactam 14.3% [34/238]), followed by cephalosporins (108/238 [45.4%]; percent resistant to Cefepime 10.5% [25/238]) (Table S3). While holding time constant, individuals with MDR‐GNRBs received an average of 18.9% (CI: 8.8, 29.1%) less treatment in the outpatient setting than individuals with non‐MDR infections. The average proportion of treatment time spent outpatient by the most commonly resistant isolates and MDR status is shown in Figure S2. We did not find evidence that MDR status modified the relationship between calendar time and proportion of time spent in the outpatient setting (*p* = .663) (Figure 2B).

## 4 DISCUSSION

We observed a low overall prevalence of GNRB and, in agreement with previous studies, a significant decline in the incidence of GNRB.^24^, ^25^ We had assumed that outpatient antibiotic treatment time would increase over time given the increasing proportion of outpatient transplants and availability of once‐daily antimicrobials (i.e., ertapenem). However, we found that the proportion of treatment time allogeneic HCT recipients with GNRBs spent outpatient declined over the 10‐year study period. A similar decline was observed among patients with similarly complex infections or pretransplant health states. In contrast, no significant decline was observed among patients with similar posttransplant complications.

Over the 10‐year study period, we found that each year, allogeneic HCT recipients with GNRBs received an average of 3.3% (95% CI: 1.6%, 5.0%) less treatment in outpatient settings than in the prior year. While surprising, what was more surprising was that we observed comparable reductions among patients with similar infection and pretransplant complications. Following adjustment for infection and pretransplant complications, the slope of the decline reduced but remained significant. These results suggest that, among patients with similar complexities, antibiotic treatment actually shifted towards inpatient care and that changes in the frequency of these complexities are unlikely the reason for the observed overall decline in outpatient care.

However, the fact that we observed a loss of significance following adjustment for posttransplant complications, suggests that the location of GNRB treatment among patients with similar posttransplant complications remained similar during our study period. It further suggests that the decline in outpatient antibiotic days may be linked to the observed increase in the frequency of posttransplant complications, specifically, inpatient and neutropenic at time of culture collection. These findings are supported by prior literature indicating that neutropenia is independently associated with hospital admission and prolonged inpatient care and the fact that, by default, patients inpatient at the time of culture collection will receive at least a portion of their treatment in an inpatient setting.^26^

While our adjusted analyses suggest that increases in posttransplant complications known to be associated with inpatient care may, in part, have driven the observed decline in outpatient treatment, we failed to see the increase in outpatient time we expected under any analysis. Given the increased health risk and costs associated with inpatient treatment, the observed trends in the location of antibiotic management remain a challenge. Future research should focus on the development and implementation of additional active interventions that could support earlier conversion to outpatient antibiotic treatment or to shorter courses of therapy among allogeneic HCT recipients with GNRBs. Additionally, given the data suggesting an increase in the proportion of posttransplant complications among patients with GNRBs, research focusing on prevention during these high‐risk periods would likely have a meaningful impact on GNRB rates overall.

Our study has multiple limitations, including limited sample size from a single transplant center. Our analysis also relied on the assumptions that all patients received 14 days of antibiotic treatment and our antibiotic start‐date was 2 days after culture collection. This is likely a conservative estimate and does not reflect changes in practice which have shifted to shorter lengths of antibiotic therapy. However, given that inpatient treatment time occurs early, shortening the length of follow‐up would only inflate the observed decline in outpatient treatment. While shortening GNRB treatment times may have resulted in fewer days of outpatient therapy, shorter treatment times are important for decreasing overall antibiotic burden. Finally, we did not have data to classify bloodstream infections as per the CDC's National Healthcare Safety Network guidelines, as cases included were either identified before the development of these definitions or just as definitions were being transitioned to include the addition of mucosal barrier injury bloodstream infections.

## 5 CONCLUSIONS

The proportion of targeted antibiotic treatment time allogeneic HCT recipients with GNRBs received care in ambulatory care settings declined over a 10‐year period. When we adjusted for posttransplant complications, the decline flattened out suggesting that treatment location decisions remained consistent for patients with similar posttransplant complications. The same, however, cannot be said for patients with similar pretransplant or GNRB complexities.

## CONFLICT OF INTERESTS

Steven A Pergam reports grant support from Global Life Technologies, Inc., participates in research trials with Chimerix, Inc. and Merck & Co., and currently participates in a clinical trial sponsored by NIAID (U01‐AI132004); vaccines for this trial are provided by Sanofi‐Aventis; all outside of this submitted work.

## AUTHOR CONTRIBUTIONS

Along with Steven Roncaioli, Margaret L Lind analyzed the data and wrote the manuscript. Amanda I Phipps and Steven A Pergam assisted in analysis generation, supported the work of Margaret L Lind and Steven Roncaioli, and provided methodologic and clinical expertise. Catherine Liu and Ania Sweet contributed clinical knowledge regarding antimicrobial stewardship and multidrug resistance definitions—their work allowed for the multidrug resistance and fluoroquinolone resistance portions of this analysis. AB conducted the laboratory resistance testing of all isolates and MS calculated the hematopoietic cell transplant specific comorbidity scores for each patient included in the analysis.

## Supporting information

Supplementary information.

## Data Availability

The datasets analyzed in this study are owned by the Fred Hutchinson Cancer Research Center and Seattle Cancer Care Alliance and, for that reason, are not publicly available. Data are, however, available from the authors upon reasonable request and with permission of Fred Hutchinson Cancer Research Center/Seattle Cancer Care Alliance.
