# Supplementary material for: Are hematopoietic cell transplant recipients with Gram‐negative bacteremia spending more time outpatient while on intravenous antibiotics? Addressing trends over 10 years at a single center
Source: Immun Inflamm Dis. 2021 Jul 21;9(4):1786–94. doi: 10.1002/iid3.486 (PMC8589361; doi:10.1002/iid3.486)
Supplement: Supplementary file 1 — Supplementary information. [file IID3-9-1786-s001.docx]

**Supplement Tables:**

| **Supplemental Table 1: Linear Trends in** Patient Complexity Factors Over Ten Years of Follow-up | | |
| --- | --- | --- |
|  | **Point Estimate** | **95% Confidence Intervals** |
| Age at Transplant | 0.03 | (-0.54, 0.60) |
| HCT-CI^a^ | -0.08 | (-0.21, 0.05) |
| Proportion Non-Hodgkin's Lymphoma | 0.00 | (-0.02, 0.01) |
| ^a^HCT-specific Comorbidity Index |  |  |

| **Supplemental Table 2:** Linear Trends in Posttransplant Complications (Post-Hoc Analysis) | | |
| --- | --- | --- |
|  | **Point Estimate** | **95% Confidence Intervals** |
| Severe Acute GVHD^a^ | 0.00 | (-0.02, 0.02) |
| Inpatient at Time of Culture Collection | 0.04 | (0.03, 0.05) |
| Neutropenic at Time of Culture Collection | 0.02 | (0.00, 0.04) |
| ^a^ Graft versus host disease score of 3 or more at time of diagnosis | | |

| **Supplemental Table 3:** Frequency of drug resistance and multi-drug resistance by resistance class within HCT recipients with GNRB | | | | |
| --- | --- | --- | --- | --- |
|  | **Number of Resistant Organisms** | **Percent of Total GNRB Infections (N = 238)** | **Number of Multi-Drug Resistant Organism** | **Percent Contribution to Total MDRO**  **(N = 79)** |
| Cephalosporins | 108 | 45.4% (39.0, 51.7%) | 66 | 83.5% (75.3, 91.8%) |
| Cefepime | 25 | 10.5% (6.9, 15.2%) | 19 | 24.1% (14.8, 36.5%) |
| Beta-lactam/beta-lactamase inhibitor^a^ | 166 | 69.7% (63.9, 75.6%) | 72 | 91.1% (84.8, 97.4%) |
| Piperacillin- Tazobactam | 34 | 14.3% (10.0, 19.4%) | 23 | 29.1% (18.8, 42.7%) |
| Fluoroquinolones | 88 | 37.0% (30.8, 43.1%) | 58 | 73.4% (63.6, 83.2%) |
| Aminoglycosides | 39 | 16.4% (11.7, 21.1%) | 32 | 40.5% (29.6, 51.4%) |
| Carbapenems | 27 | 11.3% (7.3, 15.4%) | 19 | 24.1% (14.6, 33.5%) |
| ^a^Penicillin/anti-pseudomonal penicillin + beta-lactamase inhibitors | | |  |  |

**Supplement Figures:**

**Supplemental Figure 1:** Smoothed proportion of GNRBs with posttransplant complications over study period

**
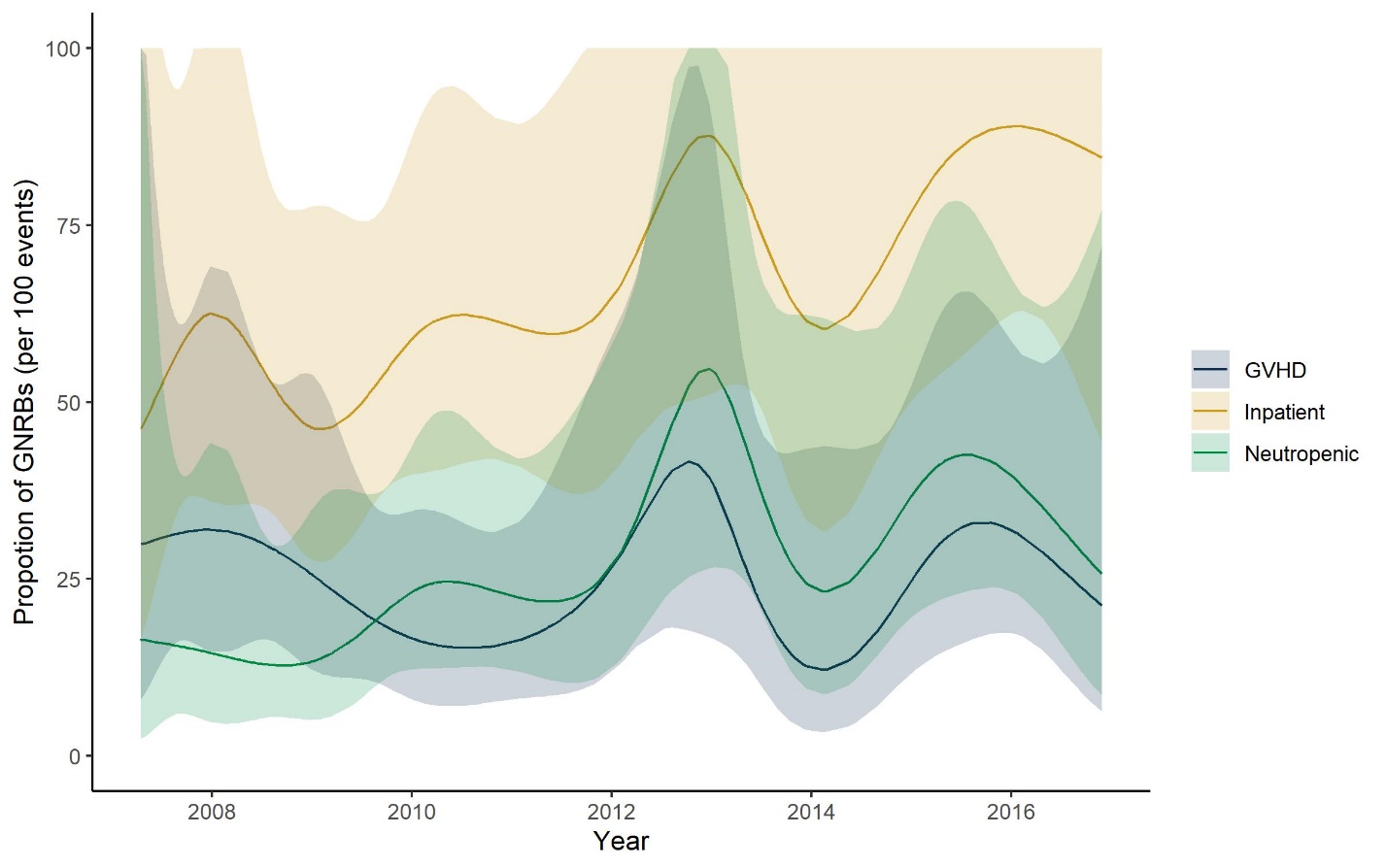
**

The smoothed proportion of gram-negative rod bacteremia (GNRB) events with posttransplant complications over the ten years of follow-up. The results were estimated using a Poisson Regression with a natural spline for time with knots at each year of study follow-up. The three posttransplant complications examined were severe acute graft verse host disease (GVHD grade 3 or more), inpatient at the time of culture collection, and neutropenic at the time of culture collection.

**Supplemental Figure 2:** Average proportion of antibiotic treatment time spent outpatient by GNRB isolated resistance profile
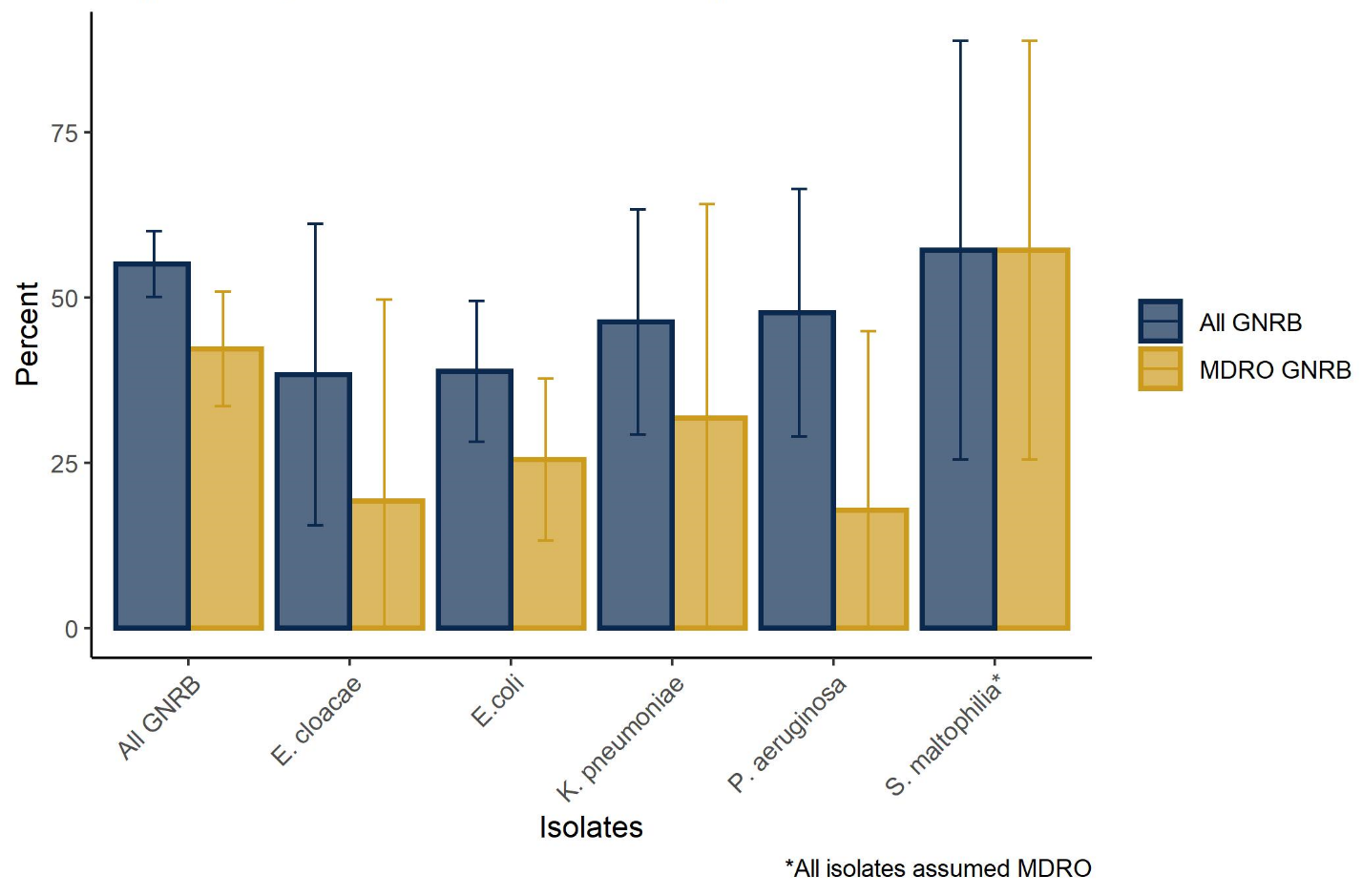


Average proportion of time allogeneic hematopoietic cell transplant (HCT) recipients with multi-drug resistant (MDR) and non-multi-drug resistant gram-negative rod bacteremia (GNRB) received targeted antibiotic treatment in ambulatory care settings by the most commonly resistant organisms within our study**.** MDR defined as cephalosporins, penicillin/anti-pseudomonal penicillin + beta-lactamase inhibitors, carbapenems, aminoglycosides, and fluoroquinolones; or any *Stenotrophomonas maltophilia* isolate. Commonly resistant organisms include: *E. cloacae (Enterobacter cloacae), E. coli (Escherichia coli), K. pneumoniae (Klebsiella pneumoniae), P. aeruginosa (Pseudomonas aeruginosa), and S. maltophilia (Stenotrophomonas maltophilia)*
